# Progress in Surgery

**Published:** 1895-01-26

**Authors:** 


### PROGRESS IN SURGERY.

### RECTAL SURGERY.

(*Continued from page* 279.)

**Hæmorrhoids.**—The best method of operating for internal hæmorrhoids is a question constantly discussed and variously answered, probably because the cautery, ligature, and Whitehead's methods are all so satisfactory. Pearce Gould[1] strongly advocates the clamp and cautery. He considers that it is the least painful method—much less painful than ligature, and that retention of urine is less likely to occur than after ligature. If done carefully and thoroughly, Pearce Gould considers there is no danger of hæmorrhage. His directions for the application are that the clamp should be screwed up as tight as possible, and with the knife of a Pacquelin's cautery the pile is to be burnt through about a quarter of an inch beyond the clamp. The stump should then be burnt down to the surface of the clamp by the same cautery at a dull red heat, and the more time is taken in thus cauterising it the more successful will be the operation. Then the clamp is very slowly unscrewed so as not to pull open the compressed and seared stump. If the operation is carried out in this way, the risk of hæmorrhage, primary or secondary, is considered nil, though, as Pearce Gould says, it can be done in such a way as to expose the patient to considerable risk of hæmorrhage. Dr. Kelsey,[2] at the New York Post Graduate Hospital, has operated on 150 cases by the clamp and cautery, and considers, with Pearce Gould, that it causes the least pain and the least vesical disturbance, and he thinks also that recovery is more rapid than after other methods. The patients are allowed to get up by the third day, and are discharged within a week. Unfortunately, the majority are never seen after the second week. He considers that nothing is gained by the ligature, but that it causes more pain, and recovery is longer; and that nothing is gained by the time taken in the performance of Whitehead's operation. Pearce Gould has known the occurrence of hæmorrhage from slipping of the ligature, but says, "If you once seal the vessels in the base of a pile thoroughly with the cautery, I have never known them to reopen and bleed." Dundore[3] recommends the ligature as the safest method of operating for internal piles, as he considers it less likely to be followed by hæmorrhage or stricture; whereas he agrees with Pearce Gould and Kelsey in thinking the clamp and cautery cause less pain, a shorter convalescence, and are less likely to be followed by retention of urine than when the ligature is used. Hæmorrhage, he considers, may very often follow the improper application of the clamp, and of course the safety of this method, as of ligature, must depend on the thoroughness of its application. He differs from Pearce Gould in thinking stricture more likely to follow the clamp than the ligature. Whitehead's operation ought, he contends, be reserved for those cases in which the entire circumference of the anus is involved, as it is followed, he thinks, by severe after effects, and could not do more than the clamp and cautery, or ligature. The hæmorrhage from Whitehead's operation may be considerable, and reflex disturbance marked, but in Whitehead's hands it has not been a dangerous operation, as he has recorded 300 cases without a death, or a single instance of secondary hæmorrhage.

Several other methods of treating piles have lately been advocated, such as excision after the use of an elastic clamp which is sewn to the stump of the pile[4]; the injection of ethereal solutions of iodoform at the base of the piles[5]; division of the internal sphincter after ligature and cauterising the piles[6]; but none of these methods commend themselves to us, and we only refer to them. Dr. Kelsey[7] says that he has spent much thought in trying to find some way of radically curing piles which, whilst satisfactory, should be free from the fear which the word operation causes. His experience with carbolic acid injection has been that, when it is radical, it is attended by more pain and risk than any of the recognised operations, and when the pain and risk are escaped it is only paliative. He has also tried multiple punctures with the galvano-cautery, as in the treatment of nævus, and with regard to the result, says by such means he can cure any case of hæmorrhoids, but the total of pain and confinement is greater than by the clamp or cautery.

**Rectal Examinations.**—Dr. Green[8] strongly recommends that anæsthesia should be more frequently employed for rectal examinations, and discusses the desirability of introducing the whole hand into the rectum, and concludes from his own experience that in selected cases manual examination of the rectum, with a hand measuring seven-and-a-half inches or less, can be practised with safety, though there is danger of rupture if the hand is larger. He refers, however, to Cripp's experiments on the dead body with a hand of this size, in which the rectum was twice ruptured, but he thinks this less likely to happen in the living.

**Anal Fissure.**—Dr. Adler[9] contends that the simple little operation commonly employed for anal fissure is not necessary in a great many cases, but that the disease can be cured by the application of a strong solution of nitrate of silver or ointments of certain formula. The ulcer must be well exposed by separation of the anal margin and cocaine applied, a proceeding which would probably cause great pain.

**Anal Tuberculosis.**—A rare form of this condition is reported by Dr. W. Kramer,[10] in which a granulation mass "the size of half an apple" was situated at the anus. It much resembled a new growth, but with a magnifying glass small greyish points were discovered in the granulations, and microscopic examination, after removal, proved the mass to be tubercular. The patient was suffering from phthisis.

[1] Clin. Journ., Sept. 5, 1894, p. 301. [2] New York Med. Journ., June, 1894, p. 750. [3] Matthews Medical Quarterly, July, 1894. [4] Ibid. [5] New York Med. Journ., July 21, 1894, p. 74. [6] Brit. Med. Jour., Epitome, Sept. 29, 1894. [7] Loc. Cit., p. 751. [8] American Medical and Surgical Bulletin, Aug. 1. [9] The Therapeutic Gazette, July 16, 1894. [10] Centr.-Bl. f Chir., April 21, 1894; and Medical Week., Aug. 31, 1894.

